# Complete genome sequences of *Aeromonas and Pseudomonas* phages as a supportive tool for development of antibacterial treatment in aquaculture

**DOI:** 10.1186/s12985-018-1113-5

**Published:** 2019-01-08

**Authors:** Joanna Kazimierczak, Ewelina Agnieszka Wójcik, Jolanta Witaszewska, Arkadiusz Guziński, Elżbieta Górecka, Małgorzata Stańczyk, Edyta Kaczorek, Andrzej Krzysztof Siwicki, Jarosław Dastych

**Affiliations:** 1 Proteon Pharmaceuticals, Lodz, Poland; 2 Department of Microbiology and Clinical Immunology, Faculty of Veterinary Medicine, University of Warmia and Mazury in Olsztyn, Olsztyn, Poland

**Keywords:** Bacteriophages, WGS, *Aeromonas hydrophila*, *Pseudomonas fluorescens*, Aquaculture

## Abstract

**Background:**

Aquaculture is the fastest growing sector of food production worldwide. However, one of the major reasons limiting its effectiveness are infectious diseases among aquatic organisms resulting in vast economic losses. Fighting such infections with chemotherapy is normally used as a rapid and effective treatment. The rise of antibiotic resistance, however, is limiting the efficacy of antibiotics and creates environmental and human safety concerns due to their massive application in the aquatic environment. Bacteriophages are an alternative solution that could be considered in order to protect fish against pathogens while minimizing the side-effects for the environment and humans. Bacteriophages kill bacteria via different mechanisms than antibiotics, and so fit nicely into the ‘novel mode of action’ concept desired for all new antibacterial agents.

**Methods:**

The bacteriophages were isolated from sewage water and characterized by RFLP, spectrum of specificity, transmission electron microscopy (TEM) and sequencing (WGS). Bioinformatics analysis of genomic data enables an in-depth characterization of phages and the choice of phages. This allows an optimised choice of phage for therapy, excluding those with toxin genes, virulence factor genes, and genes responsible for lysogeny.

**Results:**

In this study, we isolated eleven new bacteriophages: seven infecting *Aeromonas* and four infecting *Pseudomonas,* which significantly increases the genomic information of *Aeromonas* and *Pseudomonas* phages. Bioinformatics analysis of genomic data, assessing the likelihood of these phages to enter the lysogenic cycle with experimental data on their specificity towards large number of bacterial field isolates representing different locations.

**Conclusions:**

From 11 newly isolated bacteriophages only 6 (25AhydR2PP, 50AhydR13PP, 60AhydR15PP, 22PfluR64PP, 67PfluR64PP, 71PfluR64PP) have a potential to be used in phage therapy due to confirmed lytic lifestyle and absence of virulence or resistance genes.

**Electronic supplementary material:**

The online version of this article (10.1186/s12985-018-1113-5) contains supplementary material, which is available to authorized users.

## Background

*Aeromonas* and *Pseudomonas* are considered one of the most important fish pathogens among the etiological agents of bacterial fish diseases with capacity of hemolysis and biofilm formation [1–3]. These aquatic bacteria are responsible for ulcer type diseases including ulcerative syndrome, bacteria haemorrhagic septicaemia, tail and fin rot, bacteria gill rot and dropsy [4–10]. The increasing prevalence of bacterial infections leads to the indiscriminate use of antimicrobials that are the most common solution in combating pathogenic microorganisms. However, in the case of aquaculture, the range of authorized antibiotics is very narrow, e.g. the Food and Drug Administration (FDA) in 2014 approved only oxytetracycline, florfenicol, and sulfadimethoxine/ormetoprim [4]. In addition, the vast majority of bacteria pathogenic to aquaculture are resistant to multiple antibiotics [11, 12]. That forces fish farmers to look for alternative solutions that allow effective protection of breeding populations. Bacteriophages (phages) are one alternative solution that could be considered as a prospective anti-microbial strategy in aquaculture [1, 13–24]. Bacteriophages are the most abundant biological entities on Earth. In almost all ecosystems that so far have been subjected to in-depth studies, it has been estimated that there are around ten phages for every microbial cell giving approximately10^30^–10^31^ phages globally [25, 26]. However, their genomic sequences represent a small fraction in public databases. Only 1956 bacteriophage genomes are currently available in the NCBI genome database [National Center for Biotechnology Information (NCBI) Genome database https://www.ncbi.nlm.nih.gov/genome . Accessed 27 Nov 2018], of which 19 are genome sequences of phages infecting *Aeromonas* (phages belonging to *Myoviridae, Podoviridae,* and *Siphovirida*e families) and 144 infecting *Pseudomonas* (phages belonging to *Myoviridae, Podoviridae, Siphoviridae, Cystoviridae, Leviviridae, Inoviridae* families and 5 unclassified). Phages useful for therapeutic purposes must meet numerous criteria, from which the most important is their lytic nature. Only whole genome sequencing (WGS) enable an in-depth characterization of phages and the choice of the phages suitable for phage therapy. In this study, we present 7 new phages infecting *Aeromonas* and 4 new phages infecting *Pseudomonas* that significantly increase the genomic information of *Aeromonas* and *Pseudomonas* phages. Furthermore, we present results of bioinformatics analysis of genomic data assessing the likelihood of these phages to enter the lysogenic cycle and experimental data on their specificity towards a large number of bacterial field isolates representing different location. Taken together these data provide an essential basis for rational selection of bacteriophages for application in phage therapy of affected populations.

## Methods

### Bacterial strains isolation

Bacterial strains were isolated from diseased rainbow trout (*Oncorhynchus mykiss*, Walbaum 1972) from 12 different freshwater farms in Poland. Samples were collected from March 2013 to August 2014 and at least 10 fish from each farm were examined. For further research samples of damaged tissues (spleen, kidney, gills) were taken. Prepared samples were diluted with sterile phosphate-buffered saline (PBS) at 1:1 (*w*/*v*). The suspensions were incubated at 27 °C for 48 h on Aeromonas selective medium (AM) (Oxoid, Basingstoke, UK) and King B Agar (Biolab, Polska). The presumed *Aeromonas* and *Pseudomonas* colonies were isolated and identified by Gram-stain, oxidase and catalase tests, standard biochemical characteristics using the API 20NE system (bio Merieux, France) and genetically by restriction fragment length polymorphism analysis (RFLP) of the 16S rRNA gene for *Aeromonas spp*. [27] and by species-specific PCR with DNA primers against a sequence of the 16S rRNA gene for *P. fluorescens* described by Scarpellini et al. [28].

Forty five strains were isolated during this study and 13 were provided by the Adam Mickiewicz University in Poznan as reference strains (both available in public repositories and clinical human isolates, Table 1.). The strains were kept at − 80 °C in LB broth supplemented with 25% glycerol. Strains were grown at 25 °C.

**Table 1:** Bacterial strains

Code	Strain	Source
R2	*Aeromonas hydrophila* 7966	Current study
R3	*Aeromonas hydrophila* 1,206,101	
R5	*Aeromonas sobria*	
R6	*Aeromonas hydrophila* 49,140	
R9	*Aeromonas hydrophila* 35,654	
R10	*Aeromonas hydrophila* 7965	
R11	*Aeromonas hydrophila* 5,247,167	
R12	*Aeromonas hydrophila* 7965 (290158)	
R13	*Aeromonas hydrophila* 49,140	
R14	*Aeromonas salmonicida* 33,658 (788242)	
R15	*Aeromonas hydrophila* 33,658	
R16	*Aeromonas hydrophila* 35,654	
R40	1B/IRS/03/13_*Aeromonas hydrophila*	
R41	2B/IRS/03/13_*Aeromonas hydrophila*	
R42	3B/IRS/03/13_*Aeromonas hydrophila*	
R43	4B/IRS/03/13_*Aeromonas hydrophila*	
R44	5B/IRS/04/13_*Aeromonas hydrophila*	
R45	6B/IRS/05/13_*Aeromonas hydrophila*	
R46	7B/IRS/05/13_*Aeromonas hydrophila*	
R48	9B/IRS/05/13_*Aeromonas hydrophila*	
R50	11B/IRS/05/13_*Aeromonas hydrophila*	
R52	13B/IRS/06/13_*Aeromonas hydrophila*	
R53	1B/IRS/04/14K_*Aeromonas hydrophila*	
R54	2B/IRS/04/14K_*Aeromonas hydrophila*	
R55	3B/IRS/04/14K_*Aeromonas hydrophila*	
R56	4B/IRS/04/14P_*Aeromonas hydrophila*	
R58	2B/UWM/03/13_*Pseudomonas fluorescens*	
R59	3B/UWM/03/13_*Aeromonas hydrophila*	
R60	4B/UWM/03/13_*Pseudomonas fluorescens*	
R61	5B/UWM/03/13_*Pseudomonas fluorescens*	
R62	6B/UWM/03/13_*Pseudomonas fluorescens*	
R63	7B/UWM/03/13_*Pseudomonas fluorescens*	
R64	8B/UWM/03/13_*Pseudomonas fluorescens*	
R65	9B/UWM/03/13_*Aeromonas hydrophila*	
R67	11B/UWM/03/13_*Aeromonas hydrophila*	
R68	13B/UWM/03/13_*Pseudomonas fluorescens*	
R71	16B/UWM/04/13_*Aeromonas hydrophila/caviae*	
R75	20B/UWM/06/13_*Aeromonas hydrophila*	
R77	22B/UWM/06/13_*Aeromonas sobria*	
R78	23B/UWM/06/13_*Aeromonas hydrophila*	
R80	25B/UWM/07/13_*Aeromonas sobria*	
R82	27B/UWM/07/13_*Aeromonas hydrophila*	
R83	28B/UWM/07/13_*Aeromonas sobria*	
R84	29B/UWM/07/13_*Pseudomonas fluorescens*	
R91	33B/UWM/08/14_*Pseudomonas fluorescens*	
R21	*Aeromonas hydrophila* RK 70363	Adam Mickiewicz University in Poznań
R22	*Aeromonas hydrophila* SK 3	
R23	*Aeromonas hydrophila* ATCC 49140	
R24	*Aeromonas hydrophila* LMG 13656	
R25	*Aeromonas hydrophila* AK 44	
R26	*Aeromonas hydrophila* ATCC 7966^T^	
R28	*Aeromonas sobria* CIP 7433^T^	
R29	*Aeromonas salmonicida* LMG 14900^T^	
R30	*Aeromonas salmonicida* LMG 3782^T^	
R31	*Aeromonas salmonicida* CDC 0434–84	
R32	*Aeromonas salmonicida* AK 46	
R33	*Aeromonas salmonicida* LMG 3780^T^	
R34	*Aeromonas salmonicida*LMG 13,450	

### Bacteriophage isolation

Bacteriophages were isolated from samples taken from the intake manifolds, representing an initial stage of the wastewater treatment process, received from the Main Sewage Treatment Plant (GOS) in Lodz or from samples of fish pond water obtained from The Stanisław Sakowicz Inland Fisheries Institute (IRS) in Olsztyn (Table 2.). The enrichment protocol was used following the procedure given by Van Twest and Kropinski [29]. Briefly, wastewater or pond water samples were filtered through a sterile filter with a pore diameter of 0.2 μm (Sartorius). Appropriate volume of purified water sample was mixed with the same volume of 2x concentrated LB broth (LabEmpire) and bacterial culture to be used in the enrichment. The enrichment cultures were incubated for 20 h at 25 °C with agitation to allow amplification of bacteriophages active against strain used in the enrichment. Following incubation, the culture was centrifuged at 4000×g, at 4 °C for 30 min and supernatant was filtered through a sterile filter (0.2 μm). The presence of lytic bacteriophages in supernatant was detected by a modified version of the double-layer method [30]. One hundred microliters of bacteriophages was mixed with 100 μl of host cells and added to four milliliters of 48 °C top agar (LB with 0.7% agar). Then, the mixture was poured onto bottom agar plate (LB with 2% agar) and incubated for 24 h. The presence of bacteriophages in the form of plaques was detected. All enrichments and phage titrations were carried out at 25 °C.

**Table 2:** Bacteriophage strains

Bacteriophage	Source	Host strain
13AhydR10PP	GOS	*Aeromonas hydrophila* 7965
14AhydR10PP	GOS	
85AhydR10PP	IRS	
25AhydR2PP	GOS	*Aeromonas hydrophila* 7966
50AhydR13PP	GOS	*Aeromonas hydrophila* 49,140
60AhydR15PP	GOS	*Aeromonas hydrophila* 33,658
62AhydR11PP	GOS	*Aeromonas hydrophila* 5,247,167
22PfluR64PP	GOS	*Pseudomonas fluorescens* 8B/UWM/03/13
67PfluR64PP	GOS	
71PfluR64PP	GOS	
98PfluR60PP	GOS	*Pseudomonas fluorescens* 4B/UWM/03/13

For purification of single bacteriophages, a single plaque was picked with a sterile Pasteur pipette and the phages were eluted with shaking for a minimum of 1.5 h in SM buffer (50 mM Tris-Cl, pH 7.5, 99 mM NaCl, 8 mM MgSO4, 0.01% gelatin). After chloroform (50 μl/ml) extraction and centrifugation (9000 x g, 5 min, room temp.), the supernatant was transferred to a new tube. Five successive plaque purifications were carried out on each phage isolate.

Phages were annotated by giving a number and abbreviation coming from the host strain name. The phage samples were stored at 4 °C.

### Host range

The host range was determined via spot test against 49 *Aeromonas spp*. and 9 *Pseudomonas sp*. strains. Bacterial lawns of each strain were made in triplicates using the double agar overlay method, on which 20 μl droplets of the phage stocks (diluted to 10^6^ plaque-forming units (PFU)/ml) were applied. After overnight incubation, the degree of lysis of the lawns was determined. The spot test was repeated three times for each phage. The following spot evaluation system was used: completely clear spot – complete bacterial lysis in the spot, turbid spot-weak bacterial lysis in the spot, no clearing – no bacterial lysis in the spot.

### Transmission electron microscopy

Visualization of bacteriophages by transmission electron microscopy were based on the method described by Maszewska et al. [31]. The high titer bacteriophage lysates were centrifuged at 24500 g for 3 h at 4 °C. Then the phages were washed twice with 5% ammonium molybdate solution (Sigma-Aldrich) pH 6.0 using the above spin conditions. The final sediments were suspended in 5% ammonium molybdate to obtain the titer of 10^11^ pfu ml^− 1^. Subsequently, one drop of the phage suspension was placed onto the formvar and carbon coated 200-mesh copper grid (Polysciences, Inc., Warrington, USA) and drained for 3 min. Then samples were negatively stained for 45 s. with 2% (*w*/*v*) phosphotungstic acid (PTA) in darkness. The ultrastructure of bacteriophages was visualized by transmission electron microscopy (TEM) with the JEM 1010 electron microscope (JOEL Ltd., Tokyo, Japan) at 80 kV in the Laboratory of Microscopic Imaging and Specialized Biological Techniques of the Faculty of Biology and Environmental Protection, University of Lodz. To examine bacteriophages samples the magnification of 60,000 to 100,000 was used.

### DNA extraction and purification

Genomic DNA were extracted using the modified method of Su et al. [32]. Briefly, bacteriophage lysates obtained after propagation on host strain was subjected to DNase I for disrupting debris of bacterial DNA. Then, for pelleting the phage particles 2 M ZnCl_2_ solution in 1:50 (v:v) was used. Next, the phage pellet was dissolved in TENS buffer (50 mM Tris-HCl, pH 8.0, 100 mM EDTA, 100 mM NaCl, 0.3% SDS) supplemented with proteinase K, which disrupted phage capsids. Deproteinated phage DNA was subjected to the extraction by the solution of phenol/chloroform/isoamyl alcohol (25:24:1).

Eluted DNA concentrations were measured using a BioSpectrometer® (Eppendorf, Hamburg, Germany) and stored at − 20 °C for further analysis.

### RFLP analysis

Digestion reaction was performed by incubating 1 μg of isolated DNA with 2.5 U of enzyme (ThermoScientific) in a final reaction volume of 20 μl at 37 °C for 2 h. The restriction fragments were separated by 1.5% agarose gel electrophoresis in TAE buffer for 2 h at 30 V and stained by the nucleic acid stain (SimplySafe™, Eurx).

### DNA sequencing

Bacteriophage genomes were sequenced by whole genome sequencing (WGS). Whole-genome shotgun sequencing was performed on the Illumina platform. DNA was sequenced using MiSeq with 2 × 300 bp reads and assumed coverage 1000 times. The actual coverage and average contig length for each bacteriophage is presented in (Additional file 1: Table S1.). The draft genomes were de novo assembled by CLC Genomic Workbench 7.5 in Genomed, Poland.

### Bioinformatic analysis

Bioinformatic analysis started with annotation of assembled genomes which was carried out automatically using DNA Master v 5.23.2 based on GeneMarks and Glimmer algorithms (tool written by Dr. Jeffrey Lawrence, the University of Pittsburgh). Then, reference sequences were found using the Basic Local Alignment Search Tool (BLASTn), NCBI which allowed to classify analyzed bacteriophages into taxonomic groups. Circular genomic maps were obtained in GenomeVx, a tool for circular chromosome visualisation (http://wolfe.ucd.ie/GenomeVx/, accessed September 2018) while linear maps were prepared in Biopython 1.72. Determination of lytic or lysogenic lifecycle was performed on the basis of PHACTS [33] as well as on careful analysis of each ORF (open reading frame) determined by DNA Master. It was performed both in BLASTp and in HHPred at web service MPI Bioinformatics Toolkit (toolkit.tuebingen.mpg.de/#/tools/hhpred; accessed February 2018) which finds remote homologs of query amino acid sequences. tRNA genes were searched using ARAGORN, a program to detect tRNA and tmRNA genes [34]. A phylogenetic tree was created based on the sequences of terminase large subunit. The evolutionary history was inferred by using the Maximum Likelihood method and JTT matrix-based model [35]. The tree with the highest log likelihood (− 11,846.74) is shown. The percentage of trees in which the associated taxa clustered together is shown next to the branches. Initial tree(s) for the heuristic search were obtained automatically by applying Neighbor-Join and BioNJ algorithms to a matrix of pairwise distances estimated using a JTT model, and then selecting the topology with superior log likelihood value. The tree is drawn to scale, with branch lengths measured in the number of substitutions per site. This analysis involved 17 amino acid sequences. There were a total of 870 positions in the final dataset. Evolutionary analyses were conducted in MEGA X [36]. Identification of antibiotic resistance genes and virulence factors was performed with help of online tools from CGE server: ResFinder 3.0 [37] and VirulenceFinder 1.5 [38]. The genome sequences of phages described in this study were deposited in GenBank under accession numbers: MH179470 – MH179480.

## Results

Thirty six *Aeromonas* spp. and nine *Pseudomonas* sp. bacterial strains were isolated from infected fish (Table 1.) and eleven bacteriophage strains were isolated from environment: seven active against *Aeromonas* spp. and four against *Pseudomonas* sp., for which 5 *Aeromonas hydrophila* and 2 *Pseudomonas fluorescens* strains were used routinely as the hosts (Table 2.).

For all of the phages we assessed the host range: in the case of the *Aeromonas* phages with a panel of 49 *Aeromonas* spp. isolates (*A. hydrophila, A. salmonicida, A. sobria*) and in the case of *Pseudomonas* phages with 9 *P. fluorescens* isolates (Table 3.). The resulting host range patterns were different for each tested phage. The broadest host range had 13AhydR10PP, 14AhydR10PP, 85AhydR10PP, 22PfluR64PP and 67PfluR64PP (42–51% of bacterial isolates were sensitive to these phages). Phage 25AhydR2PP had the narrowest host range with only 4/49 (8%) of bacterial isolates being sensitive to it. Overall, these phages showed different but complementary host ranges.

**Table 3:**
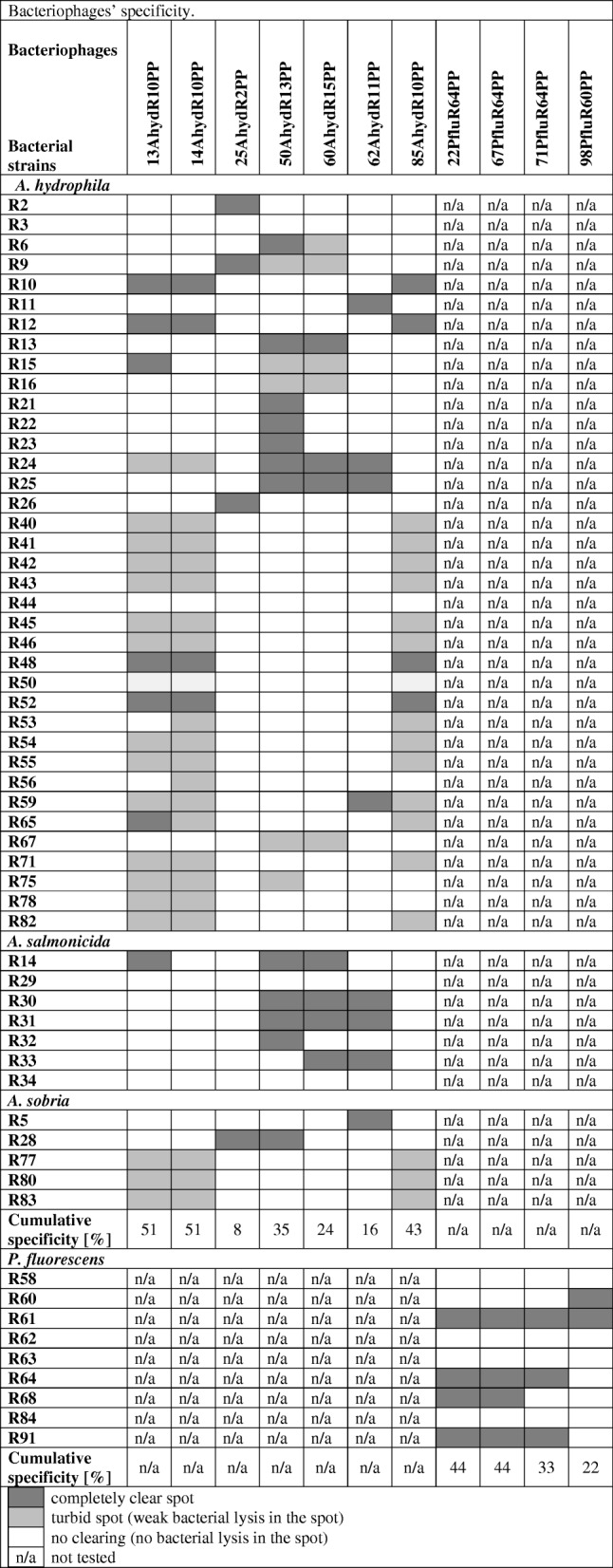
Bacteriophages’ specificity

**Fig. 1:**
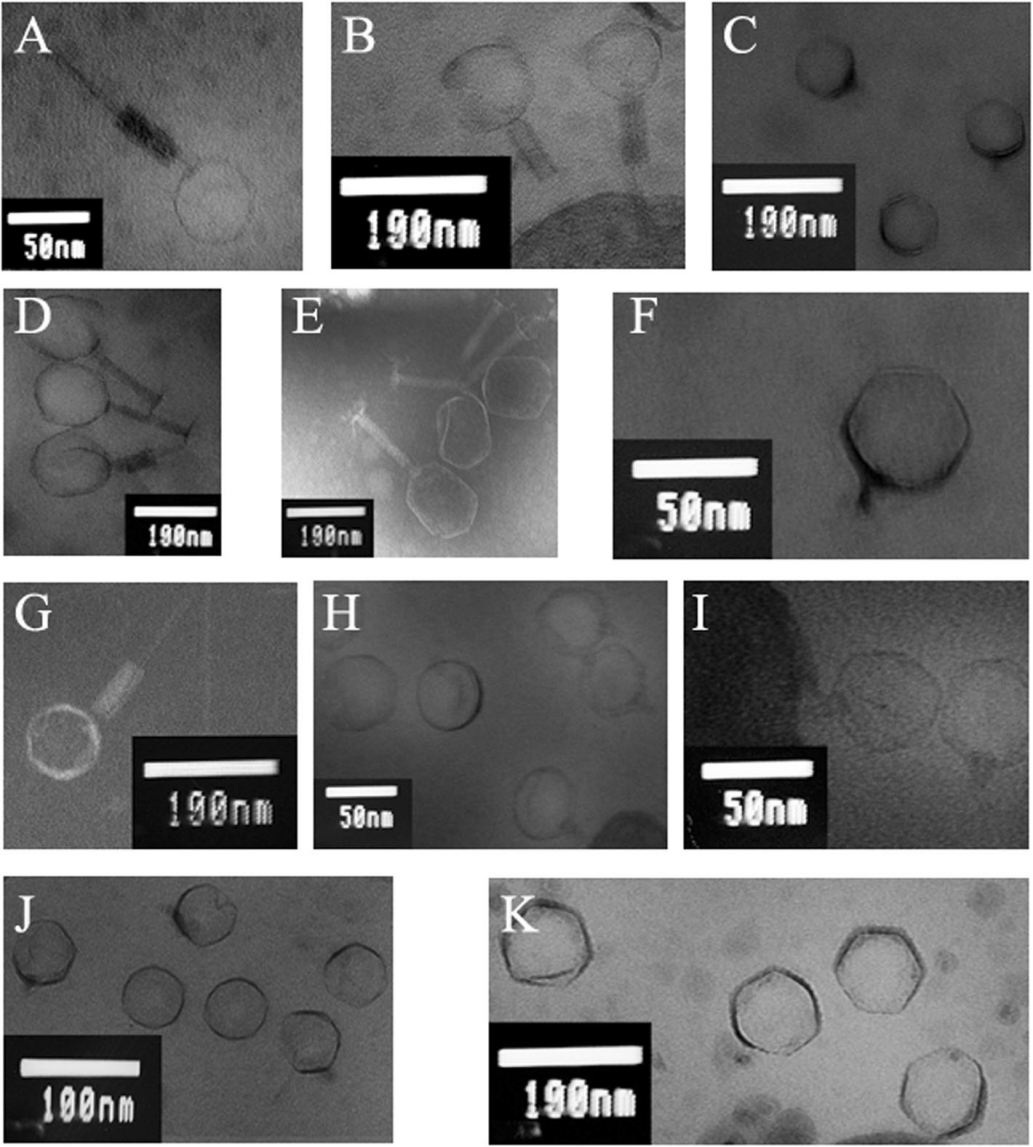
TEM micrographs of *Aeromonas* phages: 13AhydR10PP (**a**, magnification 100,000x), 14AhydR10PP (**b**, magnification 60,000x), 25AhydR2PP (**c**, magnification 60,000x), 50AhydR13PP (**d**, magnification 60,000x), 60AhydR15PP (**e**, magnification 60,000x), 62AhydR11PP (**f**, magnification 100,000x), 85AhydR10PP (**g**, magnification 60,000x), and *Pseudomonas* phages: 22PfluR64PP (**h**, magnification 100,000x), 67PfluR64PP (**i**, magnification 100,000x), 71PfluR64PP (**j**, magnification 60,000x), 98PfluR60PP (**k**, magnification 60,000x)

Visualization of bacteriophages by transmission electron microscopy showed that the tested bacteriophages 13AhydR10PP, 14AhydR10PP, 50AhydR13PP, 60AhydR15PP, 85AhydR10PP consisted of a polyhedral head and tail, which allowed classifying them in the order *Caudovirales.* In addition those phages were found to have a contracted tail characteristic for viruses belonging to the family *Myoviridae*. In contrary, phages 22PfluR64PP, 25AhydR2PP, 62AhydR11PP, 67PfluR64PP, 71PfluR64PP, 98PfluR60PP consisted of a polyhedral head and very short tail characteristic for viruses belonging to the family *Podoviridae* (Fig. 1).

**Fig. 2:**
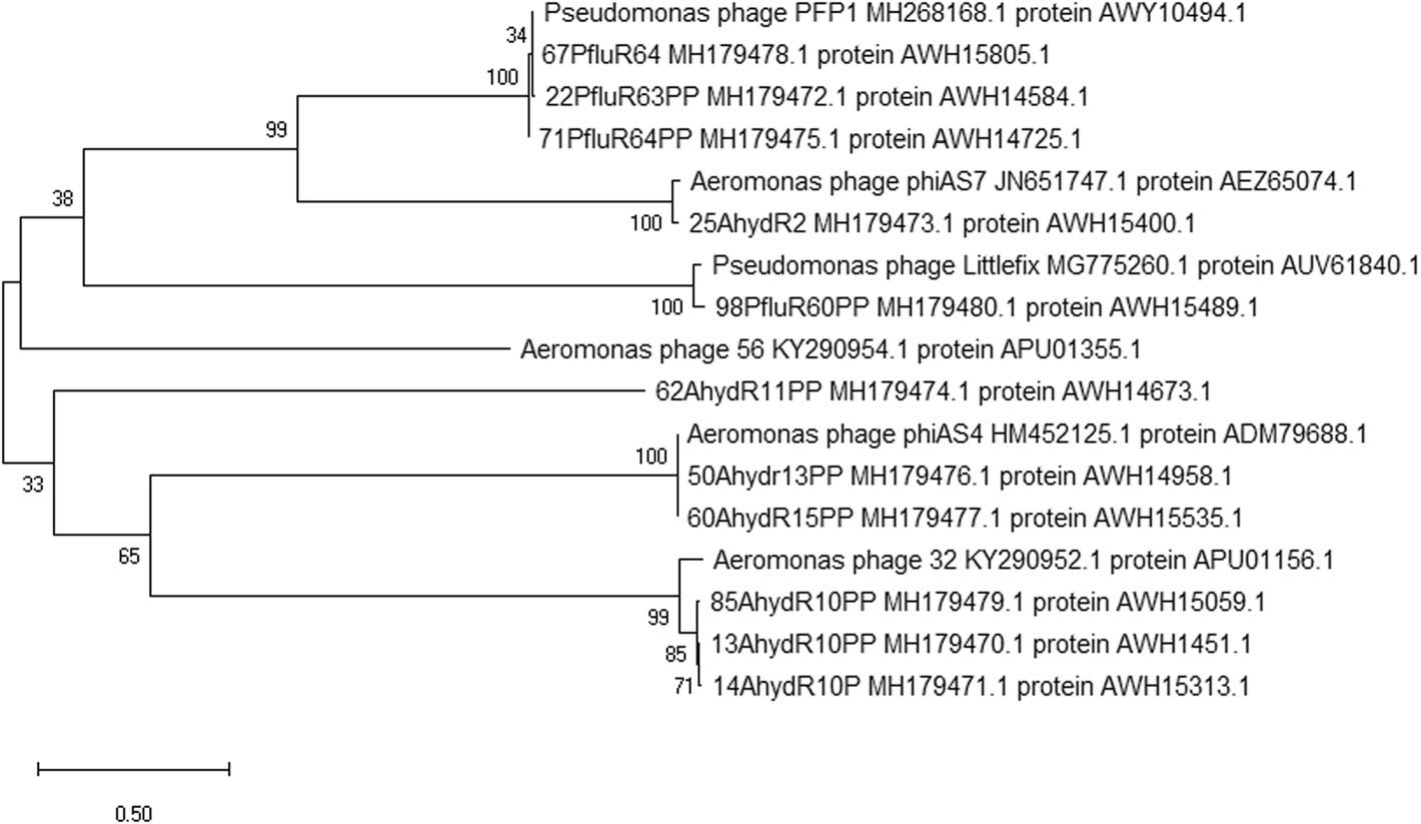
Phylogenetic tree of phage genomes (phages described in this study together with the reference strains)

Afterwards, isolation of DNA and restrictive analysis with enzymes: *Ssp*I and *Eco*RI were carried out. Obtained restriction profiles (Additional file 2: Figure S1.) allowed for the definition of initial genetic characteristics of the bacteriophages. Subsequently, after NGS sequencing (Additional file 1: Table S1.), a detailed genetic analysis of bacteriophages was performed (Table 4.). It was found that phages 13AhydR10PP, 14AhydR10PP and 85AhydR10PP possess genome sizes about 47–48 kbp and belong to double-stranded DNA viruses of *Myoviridae* family with circular genomes. They are homologues of bacterial viruses: *Aeromonas* phage 32, *Aeromonas* phage Asp37, *Aeromonas* phage 3, *Aeromonas* phage Ahp2 and *Aeromonas* phage 59.1. Moreover, their lifestyles were classified as lysogenic after conducting bioinformatic analysis, i.e. analysis of presence of genes coding for certain characteristic proteins (such as integrases or resolvases) among their ORFs and analysis of their amino acid sequences in PHACTS. They are highly similar to each other, with 96% query cover and 96% identity between 13AhydR10PP and 14AhydR10PP and 78% query cover and 89% identity between 13AhydR10PP or 14AhydR10PP and 85AhydR10PP. Phages 50AhydR13PP and 60AhydR15PP were also classified to *Myoviridae* family (*Caudovirales* order), containing linear double-stranded DNA (with circularly permuted genome) in size of approximately 165 kbp, but showing high similarity to the lytic *Myoviridae* bacteriophages specific against many bacteria from *Aeromonas* sp. They are highly similar to each other, with 94% query cover and 97% identity. PHACTS classified both of them as confidently lytic. Unclassified phage 62AhydR11PP with genome size of about 44 kbp showed low similarity with Aeromonas-infecting phages of *Myoviridae* family. It is also similar to the group of viruses with PLPE-like virion morphology. However TEM analysis allowed to classify this phage to *Podoviridae* family. Careful analysis of its ORFs allowed for the classification of this phage as probably lysogenic. Phages 25AhydR2PP, 22PfluR64PP, 67PfluR64PP and 71PfluR64PP belong to *Caudovirales, Podoviridae, Autographivirinae* with short, unshrinkable tails and icosaedral capsid containing linear double-stranded DNA of approximately 40 kbp in size. They showed a high similarity to lytic bacteriophages of T7 group specific to bacteria of the *Aeromonas* and *Pseudomonas* sp. Genome representations of these phages are linear with direct terminal repeats (DTRs). Among their ORFs no proteins responsible for lysogeny were found. Therefore, it was assumed that they exhibit lytic lifestyle. Phage 98PfluR60PP with genome size about 74 kbp has one reference genome in the NCBI database, i.e. *Pseudomonas* phage Littlefix active against *Pseudomonas* sp. It is classified into *Podoviridae* family, however with ORFs showing no or very little similarity to any known phage proteins therefore it was impossible to classify the genome of 98PfluR60PP as lytic or lysogenic on the basis of current knowledge. Labeled genetic maps (linear or circular depending on the genome) of all analyzed phages are presented in (Additional file 3: Figure S2, Additional file 4: Figure S3, Additional file 5: Figure S4, Additional file 6: Figure S5, Additional file 7: Figure S6, Additional file 8: Figure S7, Additional file 9: Figure S8, Additional file 10: Figure S9, Additional file 11: Figure S10, Additional file 12: Figure S11 and Additional file 13: Figure S12.). Afterwards, all genomes were subjected to phylogenetic analysis (Fig. 2.). The related phages can be divided into the following groups, along with the systematic classification given by GeneBank: Gr.1: 22PfluR63PP, 67PfluR64PP, 71PfluR64PP with the reference strain *Pseudomonas* phage PFP1, belong to the genus T7virus, Gr.2: 13AhydR10PP, 14AhydR10PP, 85AhydR10PP with the reference strain Aeromonas phage 32 are most likely to belong to the *Myoviridae* family, Gr.3: 25AhydR2PP together with the reference strain *Aeromonas* phage phiAS7 belong to the sub-family *Autographivirinae*, Gr.4: 98PfluR60PP with the reference strain *Pseudomonas* phage Littlefix belong to the *Podoviridae* family, Gr.5: 50AhydR13PP, 60AhydR15PP with the reference strain *Aeromonas* phage phiAS4 belong to the genus unclassified Secunda5virus.

**Table 4:** Genomic features of bacteriophages

Features	*A. hydrophila* bacteriophages							*P. fluorescens* bacteriophages			
	13AhydR10PP	14AhydR10PP	85AhydR10PP	25AhydR2PP	50AhydR13PP	60AhydR15PP	62AhydR11PP	22PfluR64PP	67PfluR64PP	71PfluR64PP	98PfluR60PP
Taxonomy	Viruses; dsDNA viruses, no RNA stage; Caudovirales; Myoviridae			Viruses; dsDNA viruses, no RNA stage; Caudovirales; Podoviridae; Autographivirinae; unclassified Autographivirinae	Viruses; dsDNA viruses, no RNA stage; Caudovirales; Myoviridae; unclassified Secunda5virus		Viruses; unclassified bacterial viruses	Viruses; dsDNA viruses, no RNA stage; Caudovirales; Podoviridae; Autographivirinae; T7virus			Viruses; dsDNA viruses, no RNA stage; Caudovirales; Podoviridae
Genome size (bp)	47,828	48,335	47,194	42,262	144,979	165,795	43,755	40,583	40,510	40,344	74,361
Predicted Lifestyle	lysogenic	lysogenic	lysogenic	lytic	lytic	lytic	probably lysogenic	lytic	lytic	lytic	not classified
No. predicted genes ()^a^()^b^	83: (74), (9)	85: (77), (8)	83: (10), (73)	52: (1), (51)	246: (205), (41)	250: (210), (40)	66: (27), (39)	53: (1), (52)	53: (1), (52)	51: (51), (0)	94: (70), (24)
Coding region (%)	90.54	91.07	90.63	93.67	92.54	92.11	92.79	92.18	92.28	92.31	88.15
G + C content (%)	59.8	57.9	59.5	55.0	41.1	41.2	57.2	59.9	60.1	59.6	42.3
No. tRNA genes	0	0	0	0	18	18	1	2	0	0	1
Accession number	MH179470	MH179471	MH179479	MH179473	MH179476	MH179477	MH179474	MH179472	MH179478	MH179475	MH179480

()^a^ + strand ()^b^- strand

X- no similarity to previously known phage families

The 62AhydR11PP phage appears to be unique. It has low similarity to phage Aeromonas 56 which is reflected on the phylogenetic tree, and they do not occur in the same clade. 62AhydR11PP bacteriophage due to the lack of similar sequences in the NCBI database and separation on the phylogenetic tree may belong to the group of phages that has not been recognized yet. Finally, all of the studied phages are deprived of any antibiotic resistance or virulence genes according to conducted bioinformatics analysis.

## Discussion

Among the entire population of phages only a few have the potential for use in phage therapy. It was previously estimated that only 50% of phages isolated from the environment are useful for therapeutic purposes [39]. However, the development of new analytical methods, including WGS, can change this proportion. Whole genome sequencing is an indispensable tool used in the study of phage biology [40–43]. WGS facilitates a detailed characterisation of phages that allows them to be classified as useful for therapeutic purposes. Phages that are promising for phage therapy should be excluded of toxin genes, virulence factor genes, and genes responsible for lysogeny [44–47]. In this study, we demonstrated 11 new bacteriophages among which 6 (25AhydR2PP, 50AhydR13PP, 60AhydR15PP, 22PfluR64PP, 67PfluR64PP, 71PfluR64PP) have a potential to be used in phage therapy due to confirmed lytic lifestyle and absence of virulence or resistance genes. At the same time, we observed (Table 3.) that most of the bacteriophages identified exhibited relatively narrow specificity to bacterial isolates. Selected *Aeromonas* phages revealed activity against only 8–35% of the whole examined *Aeromonas* collection but at the same time shows specificity towards *A. hydrophila*, *A. salmonicida* and *A. sobria*. This characteristic is contrary to previously described broad host range *Aeromonas* phages that act against only one *Aeromonas* species [13]. Narrow specificity of selected phages might create an obstacle in the development of effective phage treatment for *Aeromonas* sp. and *Pseudomonas* sp. infections. One potential way to overcome this challenge would be the creation of a multicomponent phage cocktail, consisting of completely characterized lytic bacteriophages. Using a mixture of bacteriophages to expand the coverage of treatment for heterogenous bacterial populations in bacteriophage therapy has shown high efficacy for many years. However, in most, if not all cases such bacteriophage mixtures do not consist of fully characterized bacteriophages [48, 49]. Selecting multiple components, that fulfil certain criteria, such as full genomic information, a lack of genes that create safety concerns, negligable risk of lysogenic lifestyle, and reproducible stability in the production environment, creates a significant challenge.

Bacteriophages isolated in this study have also significantly increased the knowledge about *Aeromonas* and *Pseudomonas* phages. In currently available genomes in the NCBI database among 19 described *Aeromonas* phages, 16 belong to *Myoviridae* (84% of whole population), only 2 to *Podoviridae* (10% of whole population) and 1 to *Siphoviridae* (6% of whole population). *Myoviridae* family members are most likely to be abundant in natural environments [13, 39, 50–52] and when compared to public databases, it is represented on a similar level in this study (71%). A comparable situation can be observed for *Podoviridae* phage population which is represented by 14% of *Aeromonas* phages in this study. However among the described collection there was one unclassified phage proving to be unique comparing in the public database. In the case of *Pseudomonas* phages, 41 genomes from the database belong to *Myoviridae* (28% of whole population), 46 belong to *Podoviridae* (32% of whole population), 44 belong to *Siphoviridae* (31% of whole population), 4 belong to *Cystoviridae* (3% of whole population), 2 belong to *Leviviridae* (1% of whole population), 2 belong to *Inoviridae* (1% of whole population) and 5 are unclassified (3% of whole population). When compared to the publicly available genomes, in the collection described in this study, only the *Podoviridae* family is represented and no other phage families were observed in the analyzed set of *Pseudomonas* phages. The summary of these findings is presented in Table 5.

**Table 5:** Taxonomy comparison of *Aeromonas* and *Pseudomonas* phage genomes from the database and from this study

Family	*Myoviridae*	*Podoviridae*	*Siphoviridae*	*Inoviridae*	*Cystoviridae*	*Leviviridae*	unclassified
*Aeromonas* phages							
Reference phages	pAh6-C, PX29, Aes012, CC2, Aes508, vB_AsaM-56, phiO18P, 25, 65, phiAS5, phiAS4, 32, Asp37, 3, Ahp2, 59.1	phiAS7, Ahp1	pIS4-A	x	x	x	x
% of population	84	10	6	x	x	x	x
Phages from this study	13AhydR10PP, 14AhydR10PP, 85AhydR10PP, 50AhydR11PP, 60AhydR13PP	25AhydR2PP	x	x	x	x	62AhydR11PP
% of population	71	14	x	x	x	x	14
*Pseudomonas* phages							
Reference phages	vB_PaeM _C2–10_Ab1, K5, phi3, vB_PsyM_KIL1, phiMK, K8, DL68, PhiPA3, vB_Pae_PS44, PS24, DL60, C11, vB_PaeM_PAO1_Ab03, vB_PaeM_C1-14_Ab28, vB_PaeM_PAO1_Ab27, SPM-1, phiPsa 374, PPpW-3, PAK_P5, PAK_P3, CHA_P1, PAK_P4, PAK_P2, PaBG, KPP12, PaP1, JG004, JG024, NH-4, Lu11, OBP, PB1, SN, 14–1, LMA2, LBL3, 201phi2–1, F8, EL, PAK_P1, KPP10	YMC11/06/C171_PPU_BP, PEV2, Andromeda, vB_PaeP_MAG4, YH30, PhiCHU, DL54, DL62, DL64, KPP21, PPPL-1, vB_PaeP_PPA-ABTNL, YH6, Pa2, vB_PaeP_PAO1_Ab05, vB_PaeP_C2–10_Ab22, phiPSA2, vB_PaeP_C2–10_Ab09, KPP25, TL, PPpW-4, phiIBB-PAA2, MPK6, MPK7, Phi-S1, AF, UFV-P2, tf, vB_Pae-TbilisiM32, vB_PaeP_p2–10_Or1, Bf7, phikF77, PT2, PT5, LUZ19, LUZ24, LKD16, 119X, phiKMV, PaP2, phiIBB-PF7A, phi15, LUZ7, LIT1, phi-2, Littlefix	KPP23, JBD44, YMC11/07/P54_PAE_BP, phi2, JBD93, JBD69, MD8, NP1, PS-1, PaMx11, PaMx28, PaMx42, PaMx74, PAE1, vB_PaeS_PM105, YMC11/02/R656, H70, LPB1, vB_PaeS_PAO1_Ab18, vB_PaeS_PAO1_Ab30, MP48, vB_PaeS_SCH_Ab26, phiPSA1, JD024, PA1KOR, JBD88a, JBD5, JBD30, JBD24, MP1412, MP42, vB_Pae-Kakheti25, PMG1, phi297, MP29, MP38, PAJU2, Yua, MP22, DMS3, M6, 73, F10, B3	Pf1, Pf3	phi2954, phi12, phi13, phi8	PRR1, PP7	04, vB_PaeM_MAG1, phiPto-bp6 g, vB_PaeP_Tr60_Ab31, PA11
% of population	28	32	31	1	3	1	3
Phages from this study	x	22PfluR64PP, 67PfluR64PP, 71PfluR64PP, 98PfluR60PP	x	x	x	x	x
% of population	x	100	x	x	x	x	x

## Conclusions

To conclude, 11 new bacteriophages were isolated and described on genomic level. Of these, only six have potential for phage therapy in aquaculture due to their proven lytic nature and their lack of antibiotic resistance and virulence genes. Four of them belong to the *Podoviridae*, while two to the *Myoviridae* family. The composition of these phages could be used as a therapeutic cocktail giving the cover of 41% of the *Aeromonas* and 44% of *Pseudomonas* pathogenic environmental isolates. Other phages described in this study should be excluded from any therapeutic cocktail composition due to identification of genes responsible for lysogenesis in their genomes.

## Additional files

Additional file 1: **Table S1.** DNA sequencing statistics. (DOCX 21 kb)

Additional file 2: **Figure S1.** RFLP-EcoRI (top) and RFLP-SspI (bottom) DNA profiles (electrophoresis in 1.5% agarose gel) of phages 13AhydR10PP (1), 14AhydR10PP (2), 22PfluR64PP (3), 25AhydR2PP (4), 50AhydR13PP (5), 60AhydR15PP (6), 62AhydR11PP (7), 67PfluR64PP (8), 71PfluR64PP (9), 85AhydR10PP (10), 98PfluR60PP (11). M-marker; the sizes of the molecular size markers are shown in bp on the left side of the figure. (PDF 1070 kb)

Additional file 3: **Figure S2.** Genetic map of 13AhydR10PP phage. (PDF 1451 kb)

Additional file 4: **Figure S3.** Genetic map of 14AhydR10PP phage. (PDF 1403 kb)

Additional file 5: **Figure S4.** Genetic map of 22PfluR64PP phage. (PDF 1374 kb)

Additional file 6: **Figure S5.** Genetic map of 25AhydR2PP phage. (PDF 1300 kb)

Additional file 7: **Figure S6.** Genetic map of 50AhydR13PP phage. (PDF 3305 kb)

Additional file 8: **Figure S7.** Genetic map of 60AhydR15PP phage. (PDF 3192 kb)

Additional file 9: **Figure S8.** Genetic map of 62AhydR11PP phage. (PDF 1329 kb)

Additional file 10: **Figure S9.** Genetic map of 67PfluR64PP phage. (PDF 1398 kb)

Additional file 11: **Figure S10.** Genetic map of 71PfluR64PP phage. (PDF 1445 kb)

Additional file 12: **Figure S11.** Genetic map of 85AhydR10PP phage. (PDF 1367 kb)

Additional file 13: **Figure S12.** Genetic map of 98PfluR60PP phage. (PDF 1239 kb)

## Abbreviations

BLASTn: Basic Local Alignment Search Tool
DTR: Direct Terminal Repeat
FDA: Food and Drug Administration
GOS: Main Sewage Treatment Plant in Lodz
IRS: The Stanisław Sakowicz Inland Fisheries Institute in Olsztyn
NCBI: National Center for Biotechnology Information
ORF: Open reading frame
PTA: Phosphotungstic acid
RFLP: Restriction fragment length polymorphism
TEM: Transmission electron microscopy
WGS: Whole genome sequencing
