# Supplementary material for: Complete genome sequences of Aeromonas and Pseudomonas phages as a supportive tool for development of antibacterial treatment in aquaculture
Source: Virol J. 2019 Jan 8;16:4. doi: 10.1186/s12985-018-1113-5 (PMC6325676; doi:10.1186/s12985-018-1113-5)
Supplement: Supplementary file 1 — Table S1. DNA sequencing statistics. (DOCX 21 kb) [file 12985_2018_1113_MOESM1_ESM.docx]

| **Table S1.** DNA sequencing statistics. | | |
| --- | --- | --- |
| **Phage** | **Actual coverage** | **Average contig length [bp]** |
| 13AhydR10PP | 2,881.39 | 136.41 |
| 14AhydR10PP | 1,962.62 | 211.51 |
| 22PfluR64PP | 2,782.95 | 206.22 |
| 25AhydR2PP | 280.84 | 150.5 |
| 50AhydR13PP | 115.34 | 181.2 |
| 60AhydR15PP | 792.98 | 112.39 |
| 62AhydR11PP | 2,970.63 | 179.1 |
| 67PfluR64PP | 2,584.65 | 220.69 |
| 71PfluR64PP | 2,388.13 | 198.87 |
| 85AhydR10PP | 689.59 | 114.5 |
| 98PfluR60PP | 253.33 | 123.8 |
