# Supplementary figures and images for: Complete genome sequences of Aeromonas and Pseudomonas phages as a supportive tool for development of antibacterial treatment in aquaculture
Source: Virol J. 2019 Jan 8;16:4. doi: 10.1186/s12985-018-1113-5 (PMC6325676; doi:10.1186/s12985-018-1113-5)

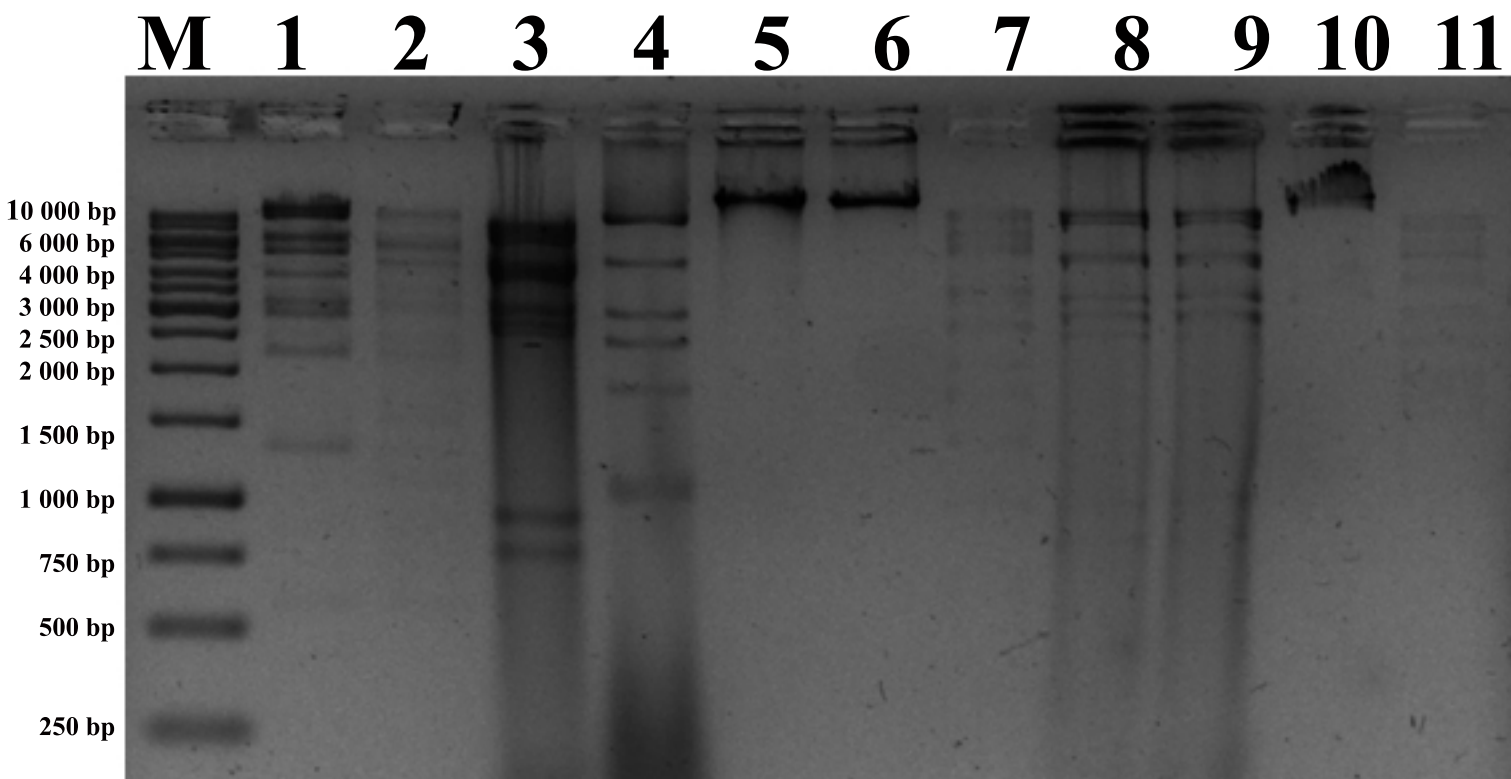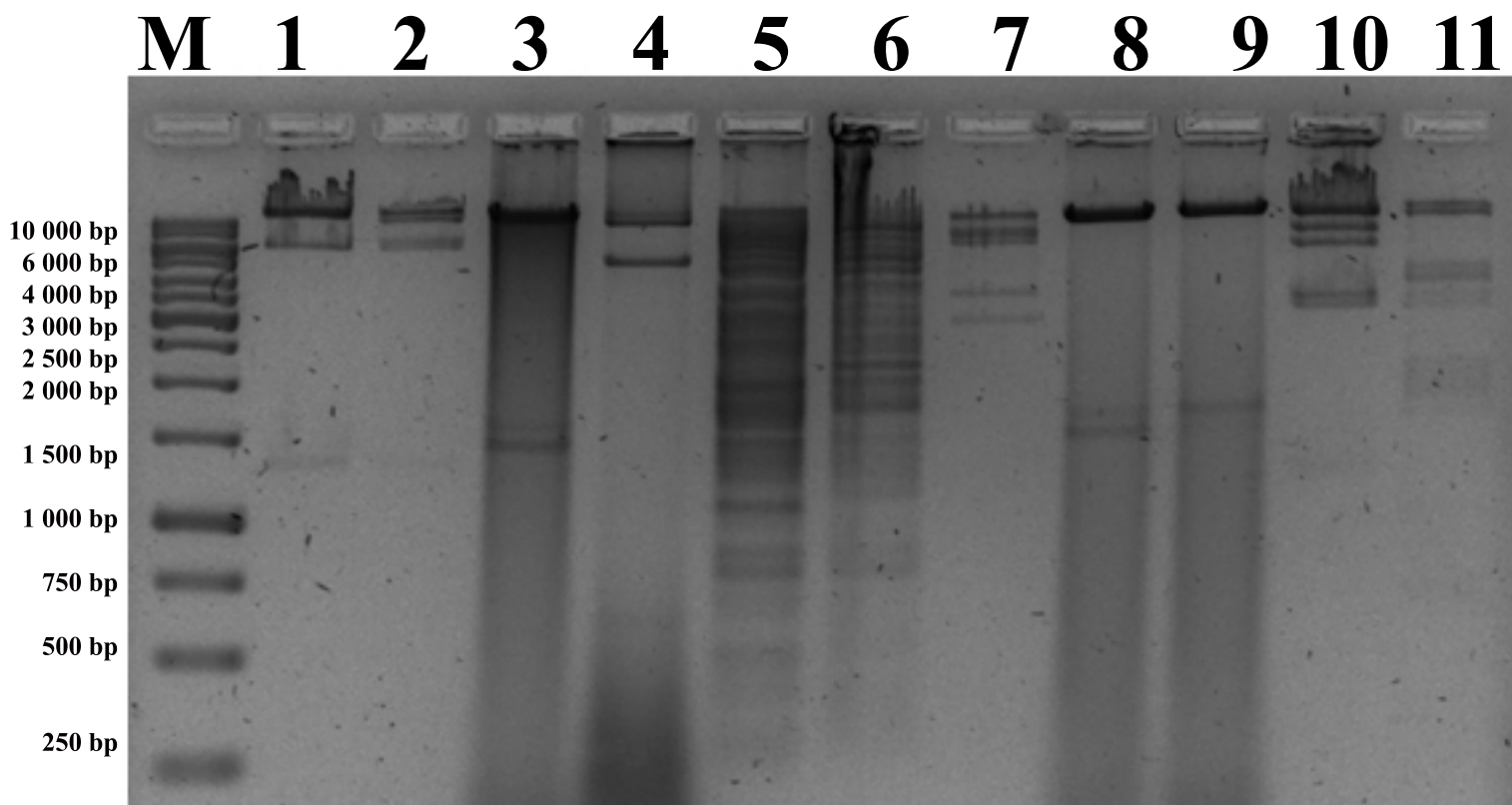

Supplement: Supplementary file 2 — Figure S1. RFLP-EcoRI (top) and RFLP-SspI (bottom) DNA profiles (electrophoresis in 1.5% agarose gel) of phages 13AhydR10PP (1), 14AhydR10PP (2), 22PfluR64PP (3), 25AhydR2PP (4), 50AhydR13PP (5), 60AhydR15PP (6), 62AhydR11PP (7), 67PfluR64PP (8), 71PfluR64PP (9), 85AhydR10PP (10), 98PfluR60PP (11). M-marker; the sizes of the molecular size markers are shown in bp on the left side of the figure. (PDF 1070 kb) [file 12985_2018_1113_MOESM2_ESM.pdf]

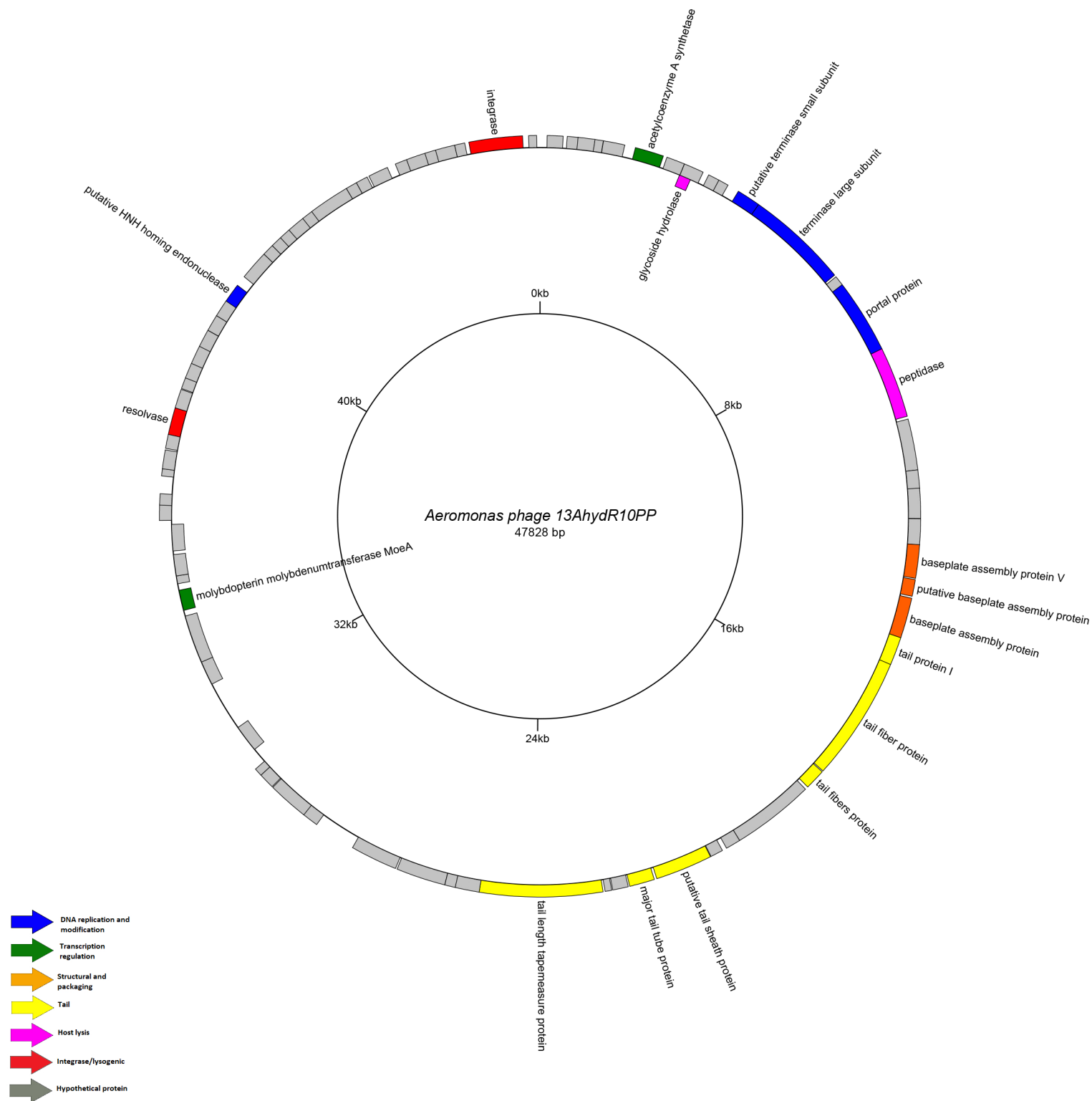

Supplement: Supplementary file 3 — Figure S2. Genetic map of 13AhydR10PP phage. (PDF 1451 kb) [file 12985_2018_1113_MOESM3_ESM.pdf]

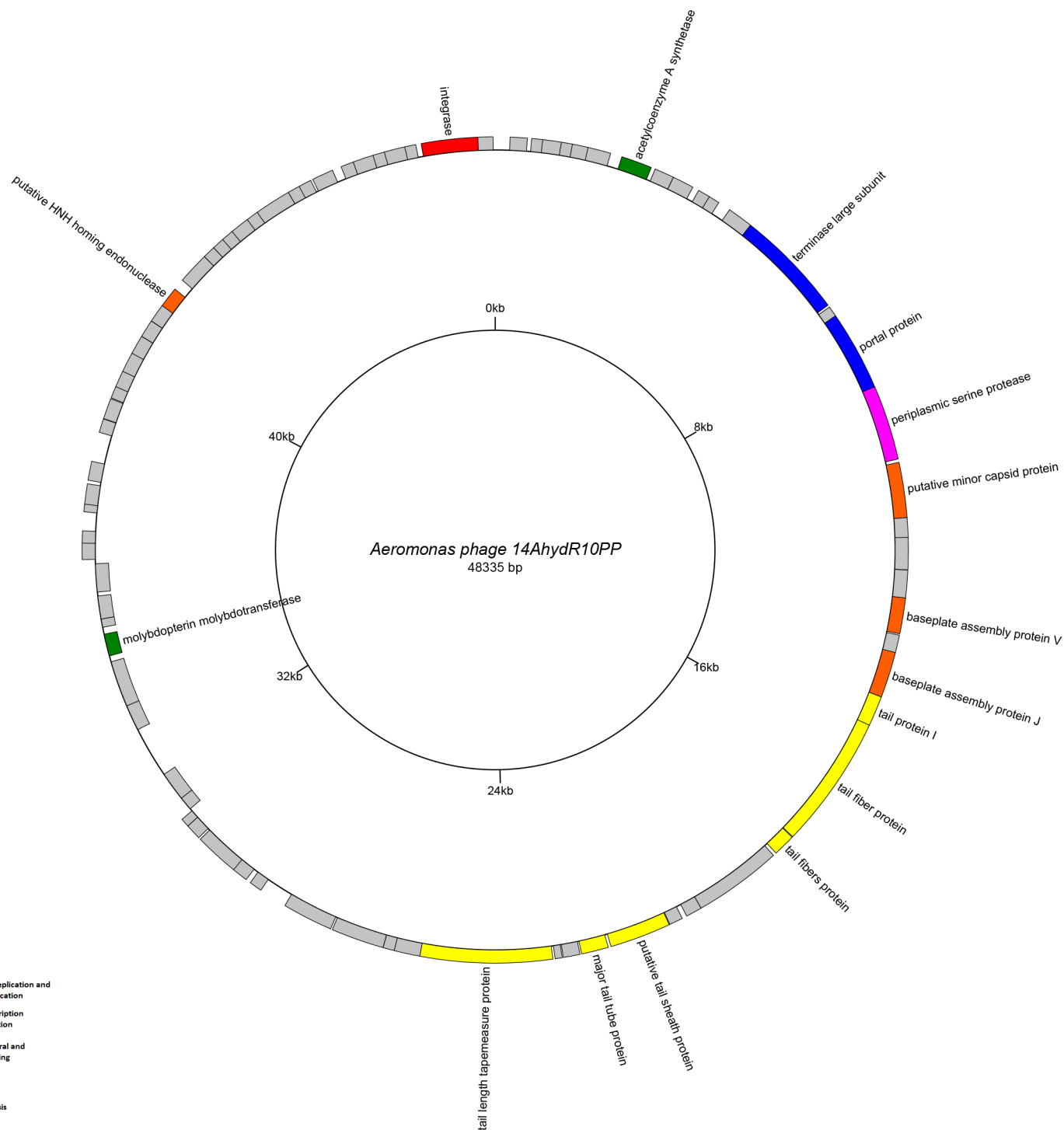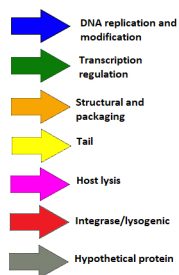

Supplement: Supplementary file 4 — Figure S3. Genetic map of 14AhydR10PP phage. (PDF 1403 kb) [file 12985_2018_1113_MOESM4_ESM.pdf]

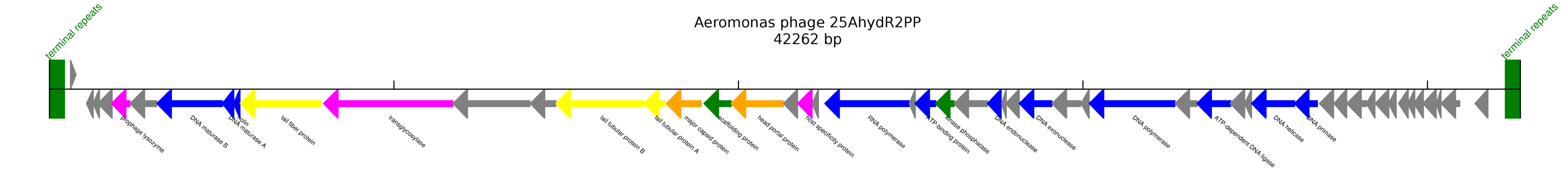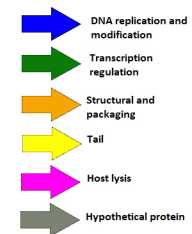

Supplement: Supplementary file 6 — Figure S5. Genetic map of 25AhydR2PP phage. (PDF 1300 kb) [file 12985_2018_1113_MOESM6_ESM.pdf]

*Aeromonas* phage 50AhydR13PP

164983 bp

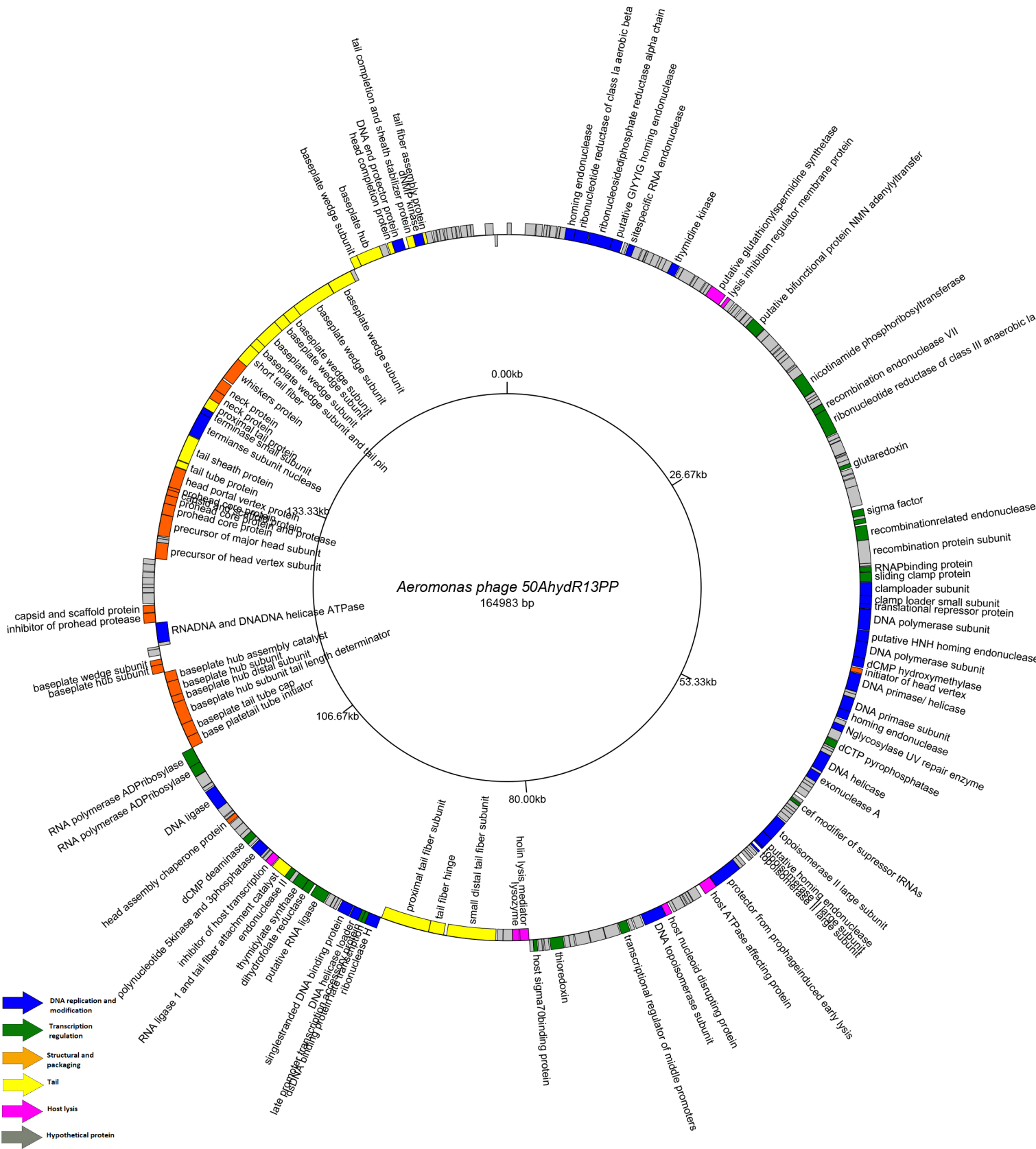

Supplement: Supplementary file 7 — Figure S6. Genetic map of 50AhydR13PP phage. (PDF 3305 kb) [file 12985_2018_1113_MOESM7_ESM.pdf]

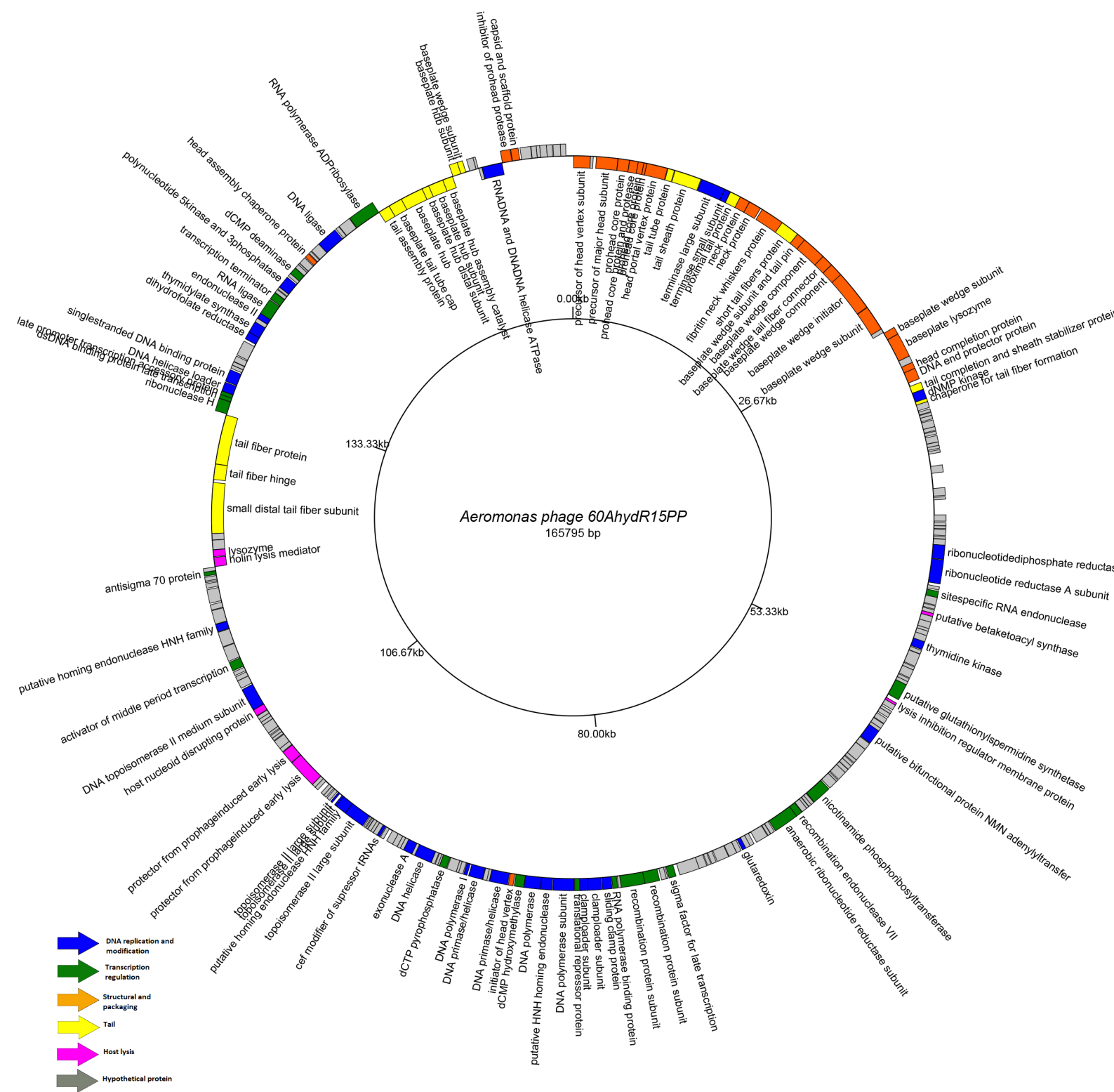

Supplement: Supplementary file 8 — Figure S7. Genetic map of 60AhydR15PP phage. (PDF 3192 kb) [file 12985_2018_1113_MOESM8_ESM.pdf]

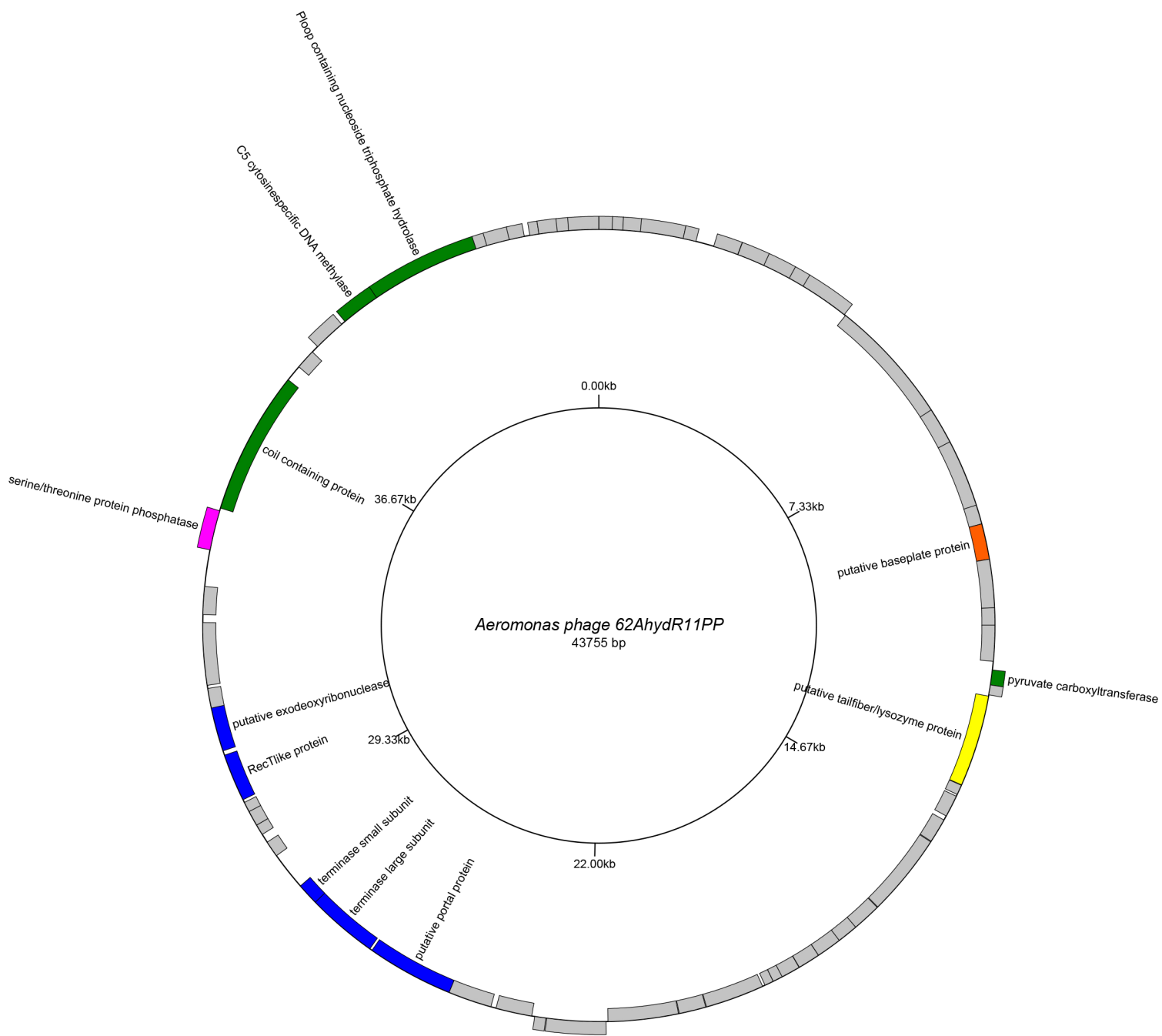

Supplement: Supplementary file 9 — Figure S8. Genetic map of 62AhydR11PP phage. (PDF 1329 kb) [file 12985_2018_1113_MOESM9_ESM.pdf]

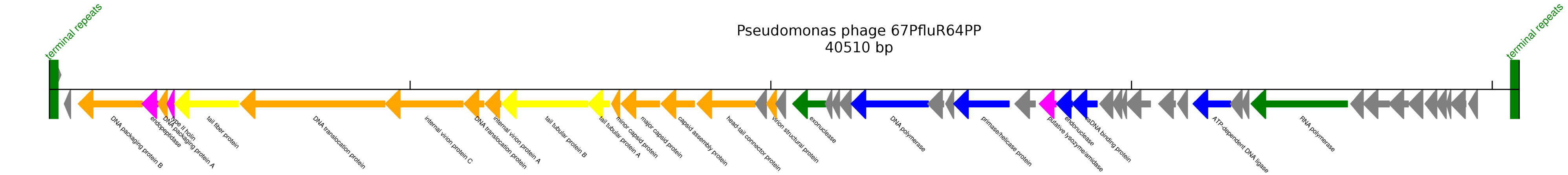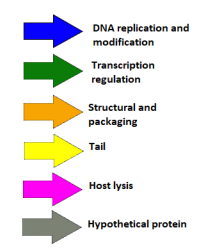

Supplement: Supplementary file 10 — Figure S9. Genetic map of 67PfluR64PP phage. (PDF 1398 kb) [file 12985_2018_1113_MOESM10_ESM.pdf]

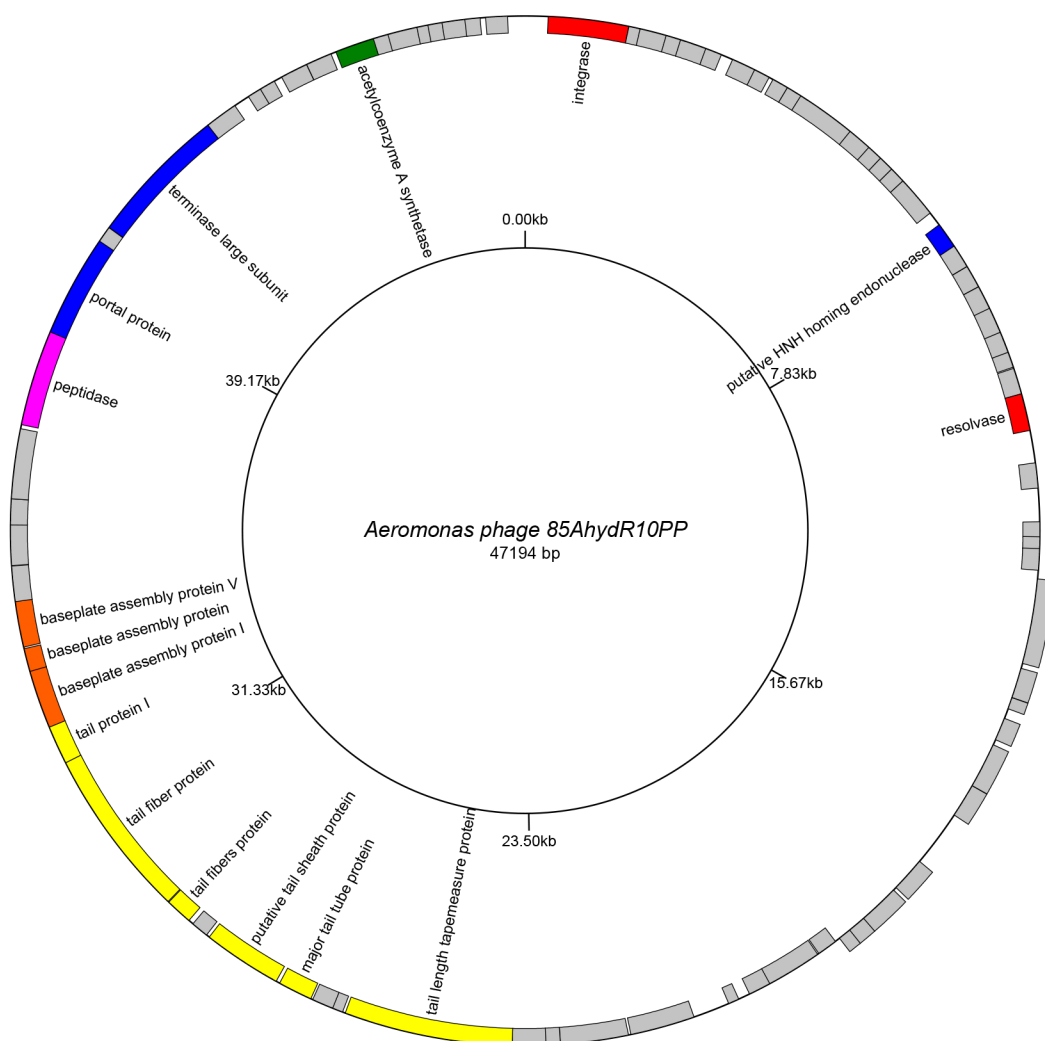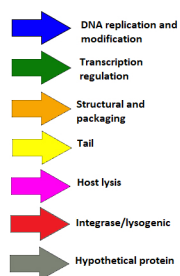

Supplement: Supplementary file 12 — Figure S11. Genetic map of 85AhydR10PP phage. (PDF 1367 kb) [file 12985_2018_1113_MOESM12_ESM.pdf]

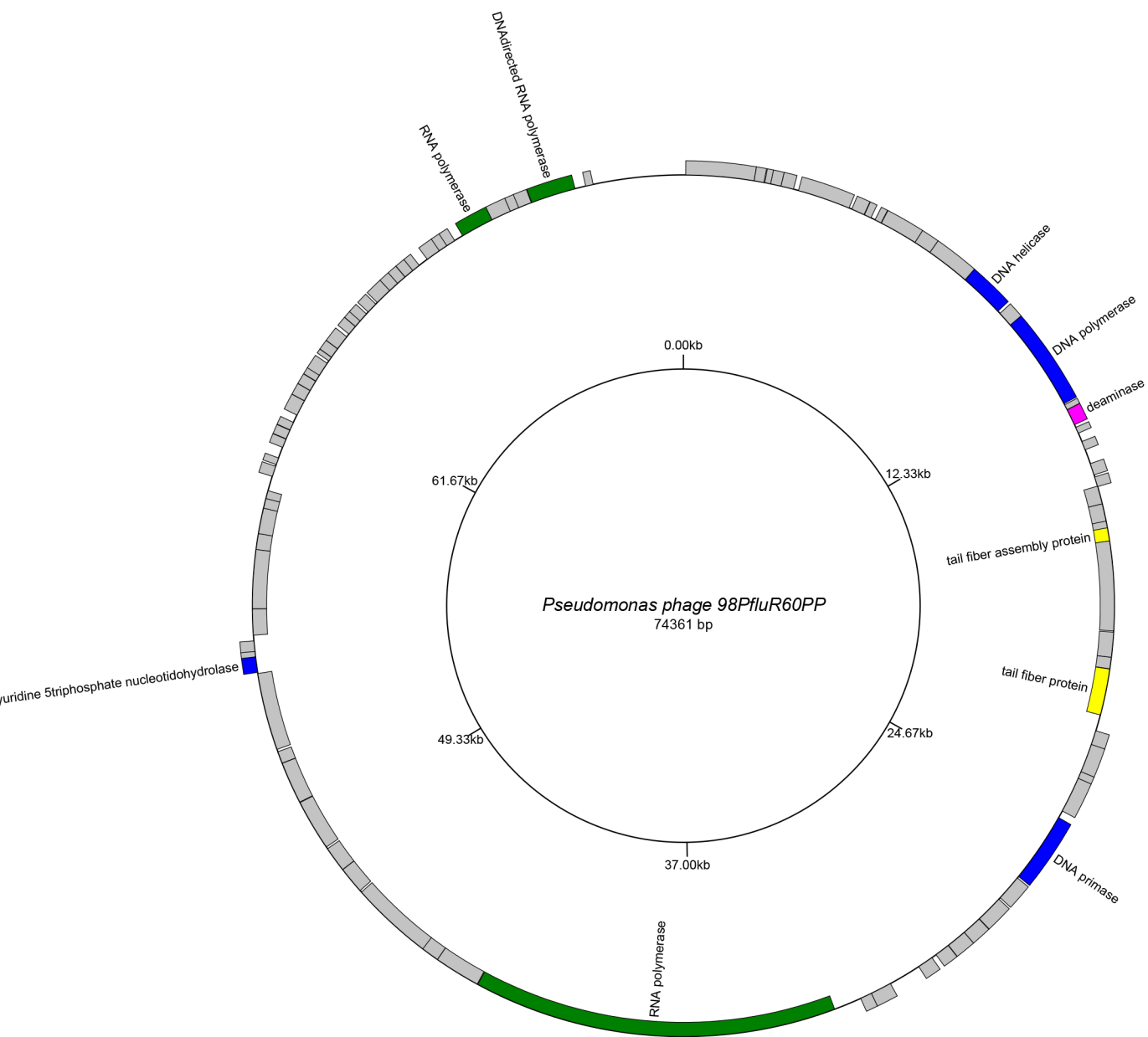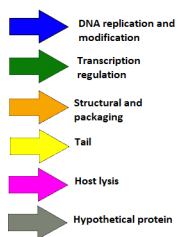

Supplement: Supplementary file 13 — Figure S12. Genetic map of 98PfluR60PP phage. (PDF 1239 kb) [file 12985_2018_1113_MOESM13_ESM.pdf]
