# Supplementary material for: Complete genome sequences of Aeromonas and Pseudomonas phages as a supportive tool for development of antibacterial treatment in aquaculture
Source: Virol J. 2019 Jan 8;16:4. doi: 10.1186/s12985-018-1113-5 (PMC6325676; doi:10.1186/s12985-018-1113-5)
Supplement: Supplementary file 5 — Figure S4. Genetic map of 22PfluR64PP phage. (PDF 1374 kb) [file 12985_2018_1113_MOESM5_ESM.pdf]

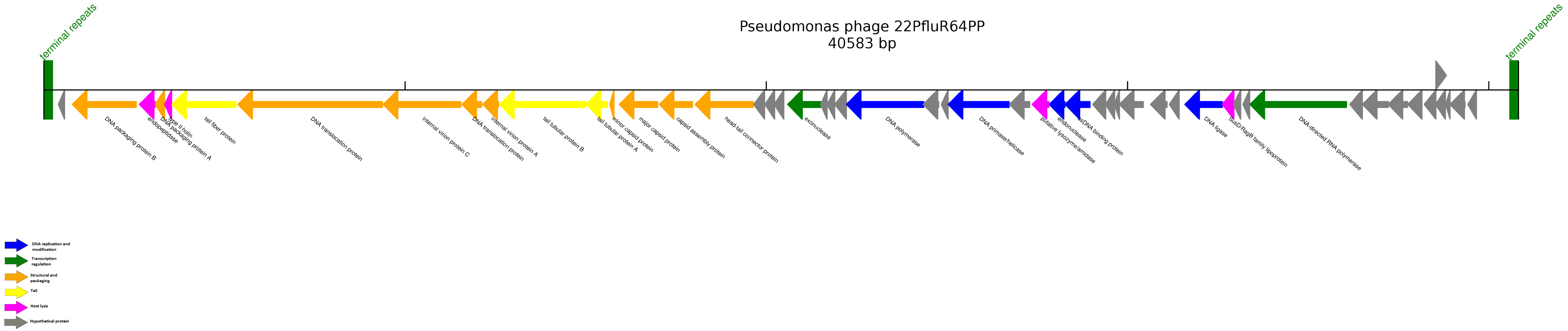
Pseudomonas phage 22PfluR64PP
40583 bp
terminal repeats
terminal repeats
DNA packaging protein B
endopeptidase
DNA packaging protein A
type II holin
tail fiber protein
DNA translocation protein
internal virion protein C
DNA translocation protein
internal virion protein A
tail tubular protein B
tail tubular protein A
minor capsid protein
major capsid protein
capsid assembly protein
head-tail connector protein
exonuclease
DNA polymerase
DNA primase/helicase
putative lysozyme/amidase
endonuclease
ssDNA binding protein
DNA ligase
SusD/RagB family lipoprotein
DNA-directed RNA polymerase
DNA replication and modification
Transcription regulation
Structural and packaging
Tail
Host lysis
Hypothetical protein
