# Supplementary material for: Complete genome sequences of Aeromonas and Pseudomonas phages as a supportive tool for development of antibacterial treatment in aquaculture
Source: Virol J. 2019 Jan 8;16:4. doi: 10.1186/s12985-018-1113-5 (PMC6325676; doi:10.1186/s12985-018-1113-5)
Supplement: Supplementary file 11 — Figure S10. Genetic map of 71PfluR64PP phage. (PDF 1445 kb) [file 12985_2018_1113_MOESM11_ESM.pdf]

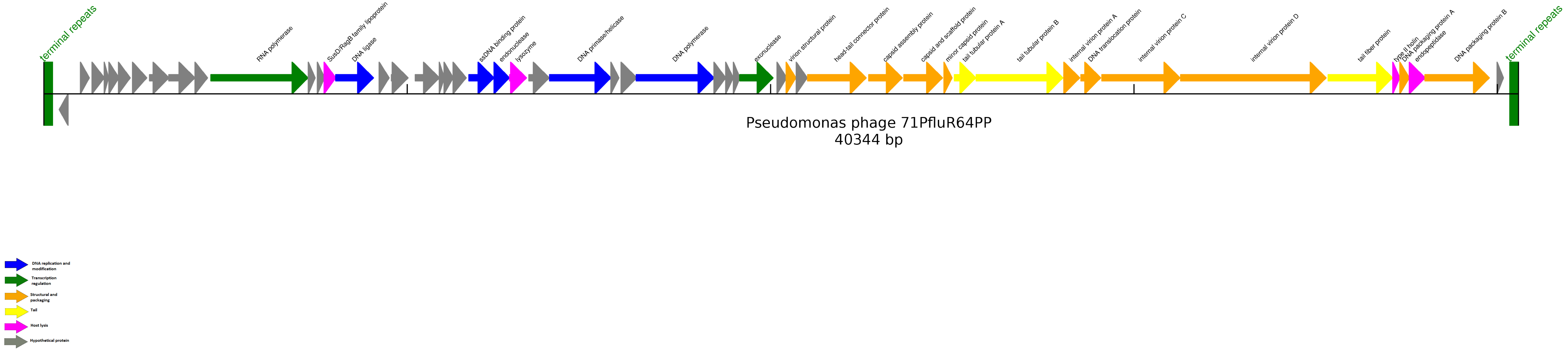

terminal repeats
RNA polymerase
SusD/RagB family lipoprotein
DNA ligase
ssDNA binding protein
endonuclease
lysozyme
DNA primase/helicase
DNA polymerase
exonuclease
virion structural protein
head-tail connector protein
capsid assembly protein
capsid and scaffold protein
minor capsid protein
tail tubular protein A
tail tubular protein B
internal virion protein A
DNA translocation protein
internal virion protein C
internal virion protein D
tail fiber protein
type II holin
DNA packaging protein A
endopeptidase
DNA packaging protein B
terminal repeats
Pseudomonas phage 71PfluR64PP
40344 bp
DNA replication and modification
Transcription regulation
Structural and packaging
Tail
Host lysis
Hypothetical protein
